# Programmatic readiness and health literacy predict the adoption of multidomain dementia prevention: a nationwide survey of Japanese municipalities

**DOI:** 10.1186/s12889-026-28068-8

**Published:** 2026-06-05

**Authors:** Yujiro Kuroda, Aya Goto, Yumi Shindo, Yohei Koyama, Kohta Suzuki

**Affiliations:** 1 Department of Health and Psychosocial Medicine, Aichi Medical University School of Medicine, Aichi, Japan; 2 Department of Global Health and Population, Harvard T.H. Chan School of Public Health, Boston, MA USA; 3 Center for Integrated Sciences and Humanities, Fukushima Medical University, Fukushima, 960-1295 Japan; 4 Center for Cultivating and Enlightenment for Healthy Longevity, Tokyo Metropolitan Institute for Geriatrics and Gerontology, Itabashi-ku, Tokyo Japan; 5 Office of Strategy and Planning, National Center for Geriatrics and Gerontology, Obu-shi, Aichi Japan; 6 Department of Public Health, Fukushima Medical University, Fukushima, Japan

**Keywords:** Dementia prevention, Implementation science, Health literacy, Municipalities, Cross-sectional study, Public health policy

## Abstract

**Background:**

Although multidomain interventions, which simultaneously address several risk factors are an evidence-based strategy for dementia prevention, their implementation status and factors driving their adoption by local governments remain unclear. This implementation gap is critical, as limited uptake on dementia prevention programs may restrict opportunities to reduce dementia risk at the population level. This study aimed to clarify the landscape of dementia prevention activities across Japanese municipalities and identify factors associated with the intention to implement comprehensive multidomain programs.

**Methods:**

A nationwide cross-sectional survey was conducted, targeting all 1,741 municipalities in Japan. A structured questionnaire was developed and used to assess the implementation of 12 World Health Organization (WHO)-recommended prevention items as well as the municipalities’ intention to adopt a multidomain program. A multinomial logistic regression analysis was performed to identify factors associated with a high adoption intention (Active Group) compared to a clear non-adoption intention (Non-Adoption Group), adjusting for municipal characteristics.

**Results:**

Of 941 included municipalities (54.0% response rate), current prevention efforts were predominantly focused on cognitive, social, and physical activities, with less emphasis on cardiovascular risk management. The strongest predictor of adoption intention was the implementation of measures to improve residents’ health literacy (relative risk ratio [RRR] = 2.8, 95% confidence interval [CI]: 1.2–6.5). Other significant factors included prior experience with multidomain programs (RRR = 1.4, 95% CI: 1.2–1.7), awareness of the WHO guidelines (RRR = 1.4, 95% CI: 1.0–2.0), and the number of currently implemented items (RRR = 1.2, 95% CI: 1.1–1.3). Structural factors such as staffing levels were not significant.

**Conclusions:**

The adoption of evidence-based dementia prevention programs by the Japanese municipalities appears to depend more on programmatic readiness and a commitment to public health communication than on structural capacity alone. These findings highlight the importance of strengthening readiness and health literacy to support municipalities in translating evidence-based dementia prevention strategies into practice and advancing population-level risk reduction.

**Supplementary Information:**

The online version contains supplementary material available at 10.1186/s12889-026-28068-8.

## Background

Dementia, a clinical syndrome, is characterized by progressive cognitive decline and includes a range of etiologies, such as Alzheimer’s disease, vascular dementia, and other neurodegenerative conditions. The increasing prevalence of dementia worldwide presents a significant public health challenge [1]. Although promising disease-modifying drugs are beginning to emerge [2, 3], their clinical integration is at the early stages and they do not offer a complete cure. Therefore, prevention has been emphasized as the primary strategy to reduce the growing societal and individual burden of the condition [4, 5]. Strong evidence now indicates that multiple modifiable risk factors across the life course contribute substantially to dementia risk. The most recent Lancet Commission on Dementia Prevention, Intervention, and Care (2024) identified 14 modifiable risk factors accounting for a considerable proportion of dementia cases worldwide [6]. In parallel, the World Health Organization (WHO) issued evidence-informed guidelines on risk reduction of cognitive decline and dementia, to support policy development and public health practice. In the present study, we focus on 12 WHO-recommended prevention items, as these provide a feasible, practical, and policy-oriented framework for assessing municipal-level factors [7]. This understanding provides a solid basis for public health initiatives aimed at reducing dementia risk by addressing these specific factors. Additionally, a growing body of research has explored or debated other risk factors besides those in the most recent Lancet Commission report, including sleep characteristics, chronic inflammation, sensory impairment, and environmental exposures [8, 9]. Although the evidence on these factors is still evolving, it highlights the dynamic and expanding nature of dementia prevention research.

Accordingly, multidomain interventions that simultaneously address multiple risk factors have become a leading preventive strategy [5, 10, 11]. Several large-scale trials—including the Finnish Geriatric Intervention Study to Prevent Cognitive Impairment and Disability (FINGER), Prevention of Dementia using Mobile phone applications (PRODEMOS), Healthy Aging Through Internet Counseling in the Elderly (HATICE), and related initiatives—have examined whether combining lifestyle modification, cognitive training, and vascular risk management can delay cognitive decline in at-risk populations [10, 12–14]. While some of these trials have demonstrated modest improvements in cognitive performance, effect sizes have been heterogeneous across populations and settings, including the use of digital tools, and participation rates in large-scale prevention programs have often been limited. These findings highlight both the potential and the challenges of translating individual-level multidomain interventions into meaningful population-level impact.

However, even when trials have demonstrated efficacy, a considerable gap exists between research findings and their incorporation into real-world practice. Knowledge of best practices for implementing dementia prevention programs at scale remains limited, presenting numerous challenges in the translation of evidence into routine care [15]. Common barriers are multi-level, including provider-level factors, like limited time and dismissive attitudes; clinic-level constraints, such as insufficient resources and workflows that prioritize acute care; and patient-level issues, like stigma and financial constraints [16–19]. Bridging this implementation gap is therefore essential to realizing the full public health impact of these interventions.

From a broad public health perspective, dementia prevention operates across multiple domains, including national policy, community environments, households, and individual behavior. Evidence from epidemiology and behavioral science suggests that individual-level interventions alone are unlikely to produce substantial shifts in population risk without complementary structural or societal change [20]. For example, secular declines in dementia prevalence reported in several high-income countries have been attributed in part to improvements in education and population-level vascular risk reduction [21]. Similarly, Japan’s long-standing salt reduction initiatives illustrate how structural cardiovascular prevention strategies can influence downstream cognitive health at scale [22]. These considerations underscore the importance of examining how preventive strategies are operationalized at the community and municipal levels, where policy translation and public engagement intersect. This multilevel perspective is increasingly reflected in contemporary frameworks advocating population-level and life-course approaches to dementia risk reduction [23]. 

In Japan, where dementia prevention is a national priority, implementation challenge is of particular importance [24, 25]. Recently, a large-scale efficacy trial, the Japan-Multimodal Intervention Trial for the Prevention of Dementia (J-MINT), was conducted [26]. As per this study, although multidomain interventions did not show a significant effect on the primary cognitive outcome in the overall group, beneficial effects were observed for individuals with a high program adherence, highlighting the importance of sustained engagement [27]. Furthermore, to bridge the gap between a resource-intensive clinical trial and real-world application, the J-MINT protocol was systematically adapted in a community setting [28], a process which demonstrated high feasibility and acceptability on a local scale [29]. These findings suggest that although community-based programs are feasible, ensuring high adherence is critical for effectiveness.

Under Japan’s Long-Term Care Insurance system, municipal governments function as meso-level actors positioned between national policy frameworks and individual residents. In this role, they translate national prevention strategies into locally tailored programs and bear legal responsibility for planning, financing, and delivering dementia-prevention and support services [30]. Disability in dementia results largely from the interaction between neurological impairments and community environments. Moreover, most people with dementia live in these communities; therefore, local structures and cultural norms strongly influence how the condition is perceived and managed [31]. From a population health perspective, the ability of municipalities to translate evidence-based dementia prevention strategies into practice depends on available resources, programmatic readiness, and capacity to communicate risk-reduction messages effectively to residents [32]. These factors are increasingly recognized as key elements in policy translation, shaping whether preventive interventions can be implemented at scale and sustained over time. Consequently, understanding the factors that determine municipal willingness and preparedness to adopt evidence-based multidomain programs is essential for building dementia-friendly communities. From an epidemiological perspective, the distribution of modifiable dementia risk factors varies substantially across populations and regions, reflecting differences in demographic structure, lifestyle patterns, and social environments. Such variability may influence population-level risk of cognitive decline as well as local priorities, perceived needs, and readiness to implement preventive interventions, underscoring the importance of examining dementia prevention efforts at the municipal level.

Accordingly, the objective of this study was to systematically describe current dementia prevention activities across Japanese municipalities and identify factors associated with municipalities’ intention to adopt multidomain dementia prevention programs. By examining how a decentralized long-term-care system operationalizes risk reduction at the municipal level, this nationwide survey aims to provide transferable insights for countries with similar preventive health mandates at regional or local authority levels, and to inform approaches for translating multidomain dementia prevention research into routine practice at scale.

## Methods

### Study design and municipalities

This study employed a cross-sectional nationwide survey design and was conducted between January and March 2025. The survey targeted all 1,741 municipalities (cities, towns, and villages) in Japan. In this study, “towns/villages” refers to the administrative classification based primarily on population size (< 50,000), rather than a direct measure of rurality. The survey did not include a separate standardized urban–rural indicator. Of the 1,741 municipalities, 941 completed the survey, yielding a response rate of 54.0%. The characteristics of the included municipalities are in Table 1.

**Table 1:** Descriptive characteristics of survey-respondent municipalities

Variable	*n*	%
Municipality size (based on the total population)		
Cities (< 100k)	150	16.3
Cities (≥ 100k)	95	10.3
Core cities	90	9.8
Towns/villages	586	63.6
Aging rate (percentage of population aged ≥ 65 years)		
<29%	235	25.5
29–34%	226	24.5
35–39%	214	23.2
≥ 40%	235	26.7
Number of staff in charge of dementia-related policies		
0–1	264	28.7
2–3	412	44.7
4–5	161	17.5
≥6	84	9.1
Number of community general support centers		
0–1	553	60.0
2–3	115	12.5
4–5	100	10.9
≥6	153	16.6

This table summarizes the characteristics of participating municipalities in the national survey. Population size categories were based on thresholds, as defined by the Ministry of Health, Labor and Welfare: “core cities” (≥ 200,000); “cities (≥ 100k)” (100,000–199,999); “cities (< 100k)” (50,000–99,999); and “towns/villages” (< 50,000). Aging rate was divided into quartiles. For dementia policy staff and community general support center variables, values of 0 were included in the “0–1” category. Percentages are based on valid responses; municipalities with missing values were excluded from the analysis.

### Survey instrument and data collection

A structured questionnaire was developed to collect data on municipal dementia prevention policies and activities. The questionnaire was adapted with permission from a nationwide baseline survey conducted in 2022, which assessed dementia prevention initiatives across Japanese municipalities [33]. Items with limited variability in response distribution were excluded, and only those deemed informative and relevant were retained. To capture implementation factors specific to multidomain interventions, new items were developed through expert consultations with researchers specializing in dementia prevention, physicians, physical therapists, and municipal officers. The final instrument included sections addressing current prevention components, organizational capacity, infrastructural and intersectoral readiness, and efforts to enhance residents’ health literacy. Although the tool did not undergo formal psychometric validation, its content was reviewed by domain experts to ensure clarity and appropriateness for the intended policy-focused audience.

To distribute the questionnaire, we asked the prefectural government official in charge of dementia policies in each of Japan’s 47 prefectures to forward the survey to relevant municipal departments within their jurisdiction. Two reminders were sent via the same prefectural channels to encourage responses. For the data collection, a dedicated website was established, allowing municipal staff to input their responses directly. Alternatively, municipalities could complete a paper version of the questionnaire and return it via fax or email.

### Variables

Primary outcome, key explanatory, and control variables included in the analyses are summarized in Table 2. Briefly, the primary outcome was municipalities’ intention to adopt a multidomain dementia prevention program. Key explanatory variables captured the extent of implementation of WHO-recommended prevention items, prior experience with multidomain programs, awareness of WHO recommendations, health literacy–related efforts, and organizational readiness. Control variables included municipality population size, aging rate, number of dementia policy staff, and number of community general support centers. Detailed definitions and measurement scales for each variable are provided in Table 2.

**Table 2:** Overview of study variables

Variable category	Variable	Brief description	Measurement / coding
Outcome	Intention to adopt a multidomain dementia prevention program	Municipal intention regarding adoption of a multidomain program	Five-category item
Key explanatory variables	Number of WHO-recommended prevention items implemented	Extent of implementation of WHO-recommended prevention components	Continuous variable (0–12)
	Prior experience with multidomain programs	Experience implementing programs combining multiple components	Binary (yes/no)
	Awareness of WHO recommendations	Level of knowledge regarding WHO dementia prevention items	Ordinal categories (5 levels)
	Health literacy–related efforts	Efforts to improve residents’ dementia-related health literacy	Mean of three 4-point items
	Infrastructure and intersectoral readiness	Infrastructure and collaboration supporting implementation	Index (mean of 3 items)
	Follow-up or post-program opportunities	Availability of follow-up opportunities	Single-item measure
	Interdepartmental coordination	Coordination across municipal departments	Single-item measure
	Community and resident engagement	Community and resident involvement	Single-item measure
Control variables	Municipality population size	Population-based administrative classification	Administrative population classification (4 groups)
	Aging rate	Proportion of residents aged ≥ 65 years	Categorized into Quartiles based on distribution
	Dementia policy staff	Number of staff in charge of dementia-related policies	Categorized into 4 groups by number of staff
	Community general support centers	Number of centers	categorized into 4 groups by number of centers

*WHO* World Health Organization

### Statistical analysis

**Fig. 1:**
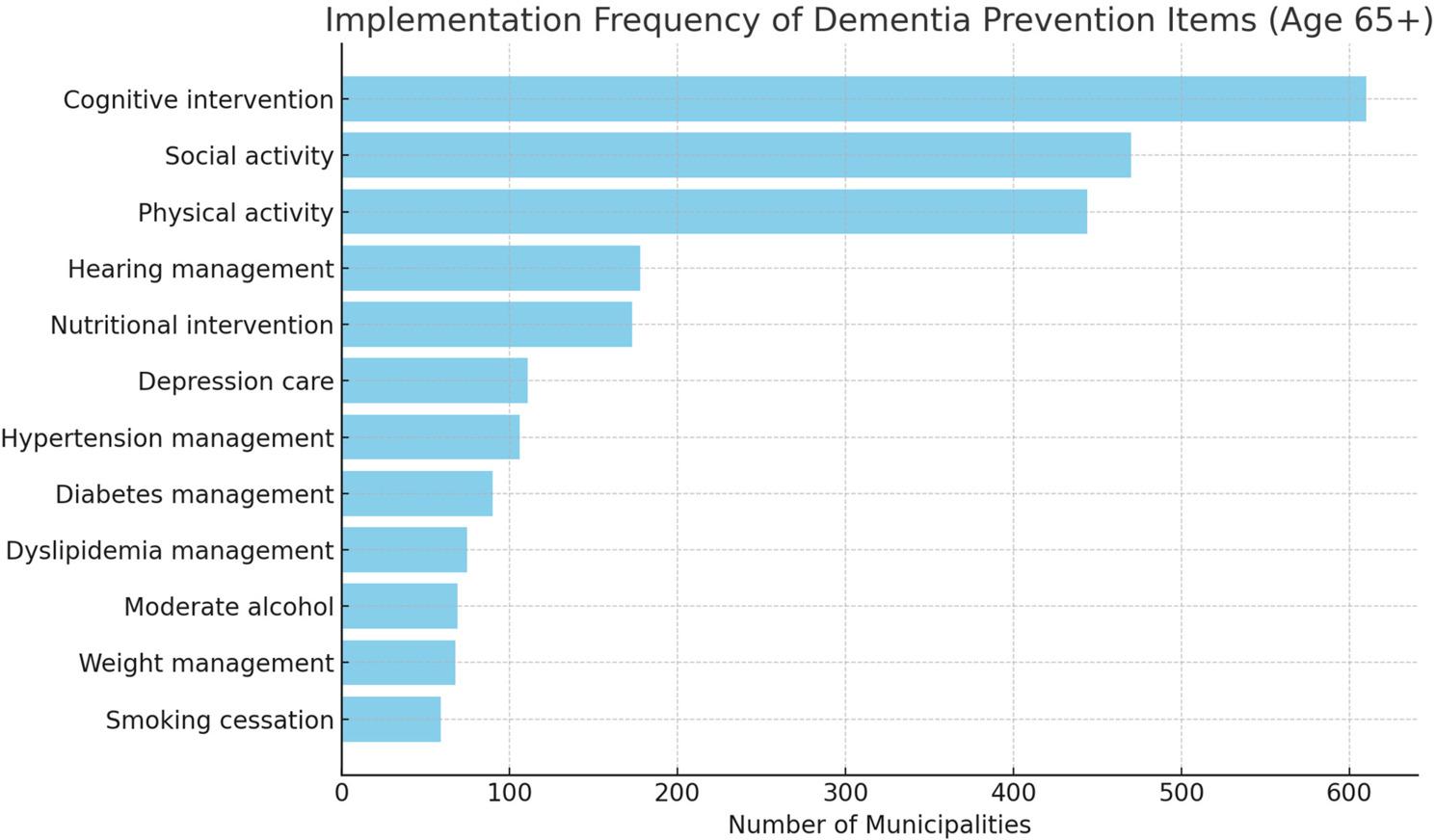
Frequency of implementation of dementia prevention items for adults aged ≥65 years. *The figure shows the number of municipalities that reported implementing each dementia prevention item for older adults (aged ≥65 years). Responses were based on a predefined list aligned with the 12 WHO-recommended domains for dementia risk reduction. Municipalities could select multiple items

Descriptive statistics were used to summarize the characteristics of the respondent municipalities and their prevention activities (Table 1, Fig. 1). For the main analysis, a multinomial logistic regression was conducted to identify factors associated with the intention to adopt a multidomain program. For the primary logistic regression analysis, municipalities that selected “Considering adoption (timeline undecided)” (n = 606) were excluded from the model. This exclusion was performed to create a more distinct and interpretable comparison between municipalities with and without a definitive positive intention. Thereby, the statistical power to identify factors associated with active adoption was increased. The remaining municipalities were then categorized into two groups for analysis. The “Active Group” (n = 110) included those that responded with “Already implementing a similar program,” “Planning to adopt immediately,” or “Planning to adopt within 2–3 years.” The “Non-Adoption Group” (n = 197) consisted of municipalities that responded with “Not planning to adopt”. The analysis calculated relative risk ratios (RRRs) and 95% confidence intervals (CIs) for the “Active Group” relative to the “Non-Adoption Group.” All models were adjusted for the four control variables listed above. In a supplementary analysis, we also conducted a multinomial logistic regression analysis, which included the “Considering adoption (timeline undecided)” group as a separate outcome category. This allowed us to examine whether key explanatory variables were associated with both active and intermediate levels of adoption intention. Results from this analysis are presented in Additional file 1.

**Fig. 2:**
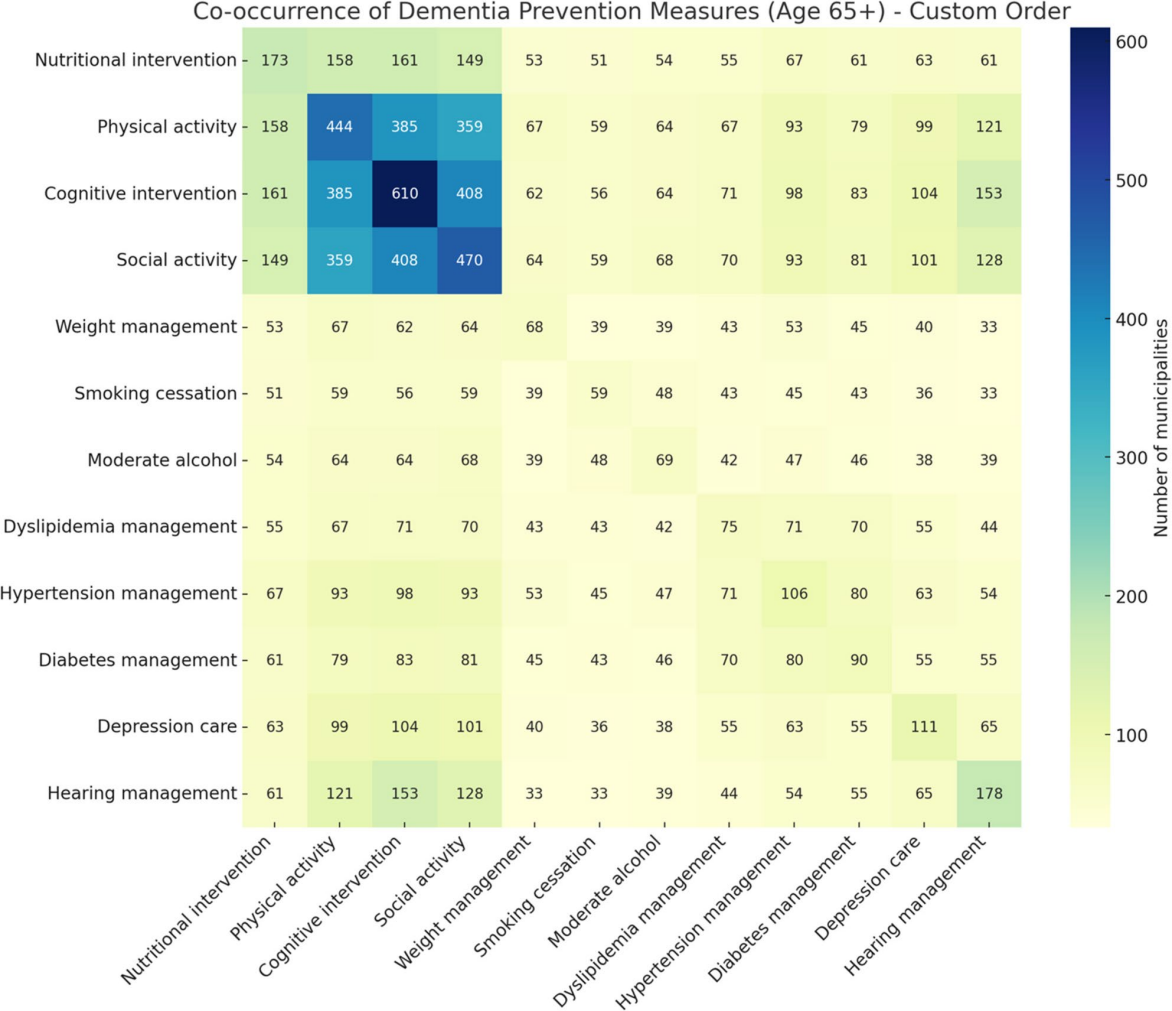
Co-occurrence matrix of dementia prevention measures implemented by municipalities (for adults aged ≥65years). *Each cell represents the number of municipalities that reported implementing both corresponding dementia prevention items. The diagonal values indicate the total number of municipalities implementing each item. The data were derived from a predefined checklist based on the 12 domains recommended by the WHO for dementia risk reduction. Multiple responses were allowed

**Fig. 3:**
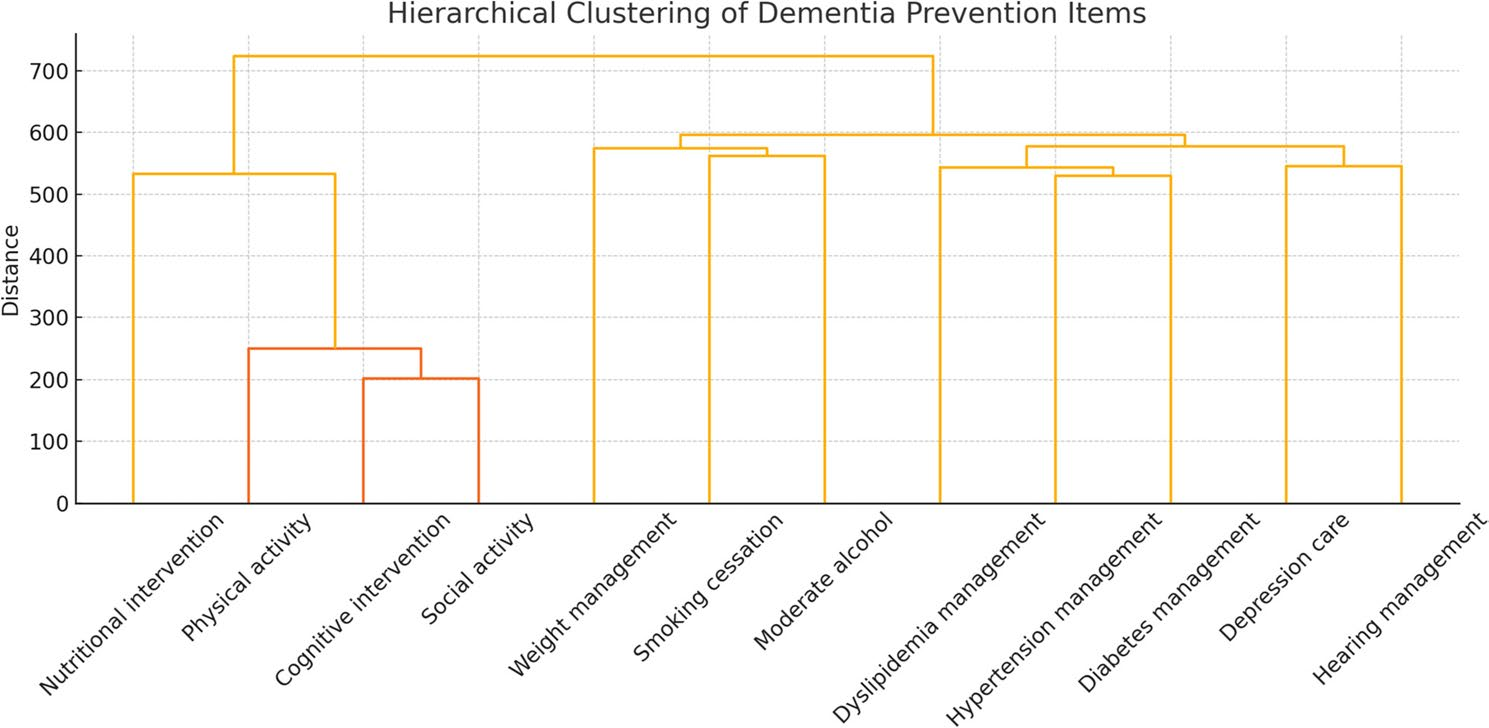
Hierarchical clustering of dementia prevention measures on co-implemented patterns. *This dendrogram illustrates the hierarchical clustering of dementia prevention items based on their co-implementation across municipalities. The clustering was performed using Ward’s method with Euclidean distance, based on a co-occurrence matrix derived from responses to a predefined WHO-aligned item list. Shorter distances between branches indicate greater similarity in implementation patterns

Exploratory analyses were performed to investigate relationships between prevention activities. A co-occurrence matrix was generated to quantify and visualize how frequently any two prevention items were implemented together, with the resulting matrix serving as the input for clustering (Fig. 2) [34]. Subsequently, a hierarchical clustering analysis using Ward’s method with Euclidean distance was conducted [35]. This analysis aimed to empirically identify natural groupings among the 12 prevention items based on their co-implementation patterns, thereby revealing underlying strategic priorities in municipal public health efforts (Fig. 3). A p-value of < 0.05 was considered statistically significant.

All statistical analyses were performed using IBM SPSS Statistics for Macintosh, Version 30.0 (IBM Corp., Armonk, NY, USA) and Python 3.10 (Python Software Foundation, Wilmington, DE, USA).

## Ethical consideration

As this survey targeted government municipalities and did not involve individual data collection, formal approval by an ethical review committee was not obtained. However, the following ethical procedures were carefully followed. A document was provided to each municipality explaining the following: (1) the protection of privacy, with assurance that published results would be aggregated to ensure anonymity; (2) that participation was entirely voluntary, with no direct benefits or disadvantage for responding or not responding; and (3) the methods for disseminating the study’s findings, including planned dissemination through peer-reviewed academic journals, presentations at national public health and dementia-related conferences, and summary reports shared with participating municipalities. Municipalities were asked to indicate their consent by checking a designated box before completing the questionnaire. Additionally, the voluntary return of the questionnaire implied consent to participate.

## Results

### Municipalities’ characteristics

A response rate of 54.0% (941 municipalities) was obtained. As shown in Table 1, most municipalities were classified as towns or villages (63.6%), followed by cities with a population of < 100,000 (16.3%). The distribution across the four aging rate categories was relatively balanced. Regarding staffing for dementia-related policies, the most common category was “2–3 staff members” (44.7%). Most municipalities (60.0%) reported having only one community general support center.

## Implementation of dementia prevention items

The frequency of implementing the 12 WHO-recommended dementia prevention items varied considerably among the included municipalities (Fig. 1). “Cognitive intervention” was the most frequently implemented (*n* = 608), followed by “Social activity” (*n* = 470) and “Physical activity” (*n* = 444). In contrast, measures targeting specific lifestyle factors, such as “Smoking cessation” (*n* = 68), “Weight management” (*n* = 78), and “Moderate alcohol use” (*n* = 82), were less frequently implemented. To provide a broad geographical context, we conducted a descriptive overview of municipalities’ adoption intention by macro area, grouping municipalities into northern (Hokkaido–Tohoku), central (Kanto–Chubu–Kinki), and southern (Chugoku–Shikoku–Kyushu–Okinawa) regions of Japan. Overall, the distribution of adoption intention was broadly similar across regions, with no marked geographical clustering. However, central regions tended to include a slightly higher number of municipalities with active or planned adoption, while northern and southern regions included a higher number considering adoption. Additionally, these patterns were exploratory and descriptive, not based on formal statistical comparison.

When examining the concurrent implementation of multiple items, the combination of “Cognitive intervention,” “Physical activity,” and “Social activity” was most commonly implemented by 108 municipalities (Table 3). The co-occurrence matrix further illustrated a strong tendency for these three items to be implemented together (Fig. 2). Hierarchical clustering analysis based on these co-implementation patterns confirmed this observation, identifying a distinct cluster comprising cognitive, physical, and social activities. This cluster was distinctly separate, from nutritional interventions to a larger grouping of measures related to vascular risk management and other lifestyle factors (Fig. 3).

**Table 3:** Most frequently implemented combinations of dementia prevention items among included municipalities

Combination	Number of municipalities
Cognitive intervention, physical activity, Social activity	108
Cognitive intervention	108
Cognitive intervention, social activity	62

This table lists the top three most frequently implemented combinations of dementia prevention items among municipalities. Combinations refer to the concurrent implementation of items. The item “Cognitive intervention” is presented separately to highlight the notable number of municipalities that reported implementing this intervention alone, without concurrent implementation with other items.

### Intention to adopt multidomain interventions

Municipalities’ intentions regarding the adoption of a multidomain dementia prevention program are presented in Table 4. In total, 76 (8.3%) municipalities reported that they were “Already implementing a similar program.” An additional 34 municipalities (3.7%) were actively planning adoption, including 20 (2.2%) that were “Planning to adopt immediately” and 14 (1.5%) that were “Planning to adopt within 2–3 years.” The largest proportion of municipalities (66.4%, *n* = 606) were “Considering adoption” within an undefined timeline. A significant minority (21.6%, *n* = 197) were “Not planning to adopt.” To support this descriptive overview, the distribution of adoption intention categories is summarized in Table 4.

**Table 4:** Intention to adopt a multidomain dementia prevention program among municipalities (*n* = 914)

	*n*	%
Already implementing a similar program	76	8.3
Planning to adopt immediately	20	2.2
Planning to adopt within 2–3 years	14	1.5
Considering adoption (timeline undecided)	606	66.4
Not planning to adopt	197	21.6

Adoption intention was self-reported by municipalities based on a five-option item. The category “Already implementing a similar program” refers to municipalities that indicated they had initiated a comparable multidomain intervention. Percentages are calculated based on valid responses.

### Factors associated with adoption intention

The multinomial logistic regression analysis identified four factors that were significantly associated with a higher intention to adopt a multidomain program (i.e., being in the “Active Group” versus “Non-Adoption Group”), after adjusting for municipal characteristics (Table 5). The strongest associated factor was the “Implementation of measures to improve residents’ health literacy” (RRR = 2.8; 95% CI, 1.2–6.5; *p* = 0.019).

**Table 5:** Factors associated with the intention to adopt a multidomain dementia prevention program

	Active^a^ vs. Non-Adoption^b^ (RRR, 95% CI)	*p*-value
Prior experience with implementing a multidomain dementia prevention program	1.4 (1.2–1.7)	< 0.001
Awareness of the WHO’s recommended dementia prevention items	1.4 (1.0–2.0)	< 0.001
Number of WHO-recommended dementia prevention items implemented (per item)	1.2 (1.1–1.3)	< 0.001
Implementation of measures to improve residents’ health literacy	2.8(1.2–6.5)	0.019
Development of infrastructure, intersectoral collaboration, and cultural foundation	0.6 (0.3–1.4)	0.255
Availability of follow-up or post-program opportunities for participants	1.3 (0.8-2.0)	0.345
Level of coordination across departments within the municipal government	1.0 (0.6–1.6)	0.883
Degree of community and resident engagement in program planning or implementation	1.1 (0.5–2.3)	0.822

*RRR *Relative risk ratio, *CI *confidence interval. Estimates were derived using multinomial logistic regression models. All models were adjusted for municipality population size, aging rate, number of staff in charge of dementia-related policies, and number of community general support centers. For the variable “Number of WHO-recommended dementia prevention items implemented,” the RRR represents the change associated with each additional item implemented. ^a^By “Active,” we refer to the number of municipalities whose responses were: “Already implementing a similar program,” “Planning to adopt immediately,” or “Planning to adopt within 2–3 years.” ^b^“Non-Adoption” refers to municipalities whose response was: “Not planning to adopt.”

Other factors significantly associated with a higher adoption intention were “Prior experience with implementing a multidomain dementia prevention program” (RRR = 1.4; 95% CI, 1.2–1.7; *p*<0.001), “Awareness of the WHO’s recommended dementia prevention items” (RRR = 1.4; 95% CI, 1.0–2.0; *p*<0.001), and the “Number of WHO-recommended dementia prevention items implemented” (RRR per additional item = 1.2; 95% CI, 1.1–1.3; *p*<0.001).

Conversely, variables such as “Development of infrastructure, intersectoral collaboration, and cultural foundation,” “Availability of follow-up or post-program opportunities,” “Level of coordination across departments,” and “Degree of community and resident engagement,” were not significantly associated with the intention to adopt a program.

To further examine potential response bias at the structural level, we compared publicly available fiscal capacity between responding and non-responding municipalities. Responding municipalities demonstrated modestly higher fiscal capacity (mean fiscal index 0.52 vs. 0.45; median 0.48 vs. 0.38), suggesting that municipalities with stronger structural resources were somewhat more likely to participate in the survey.

### Supplementary analysis including the “Considering adoption” group

In the supplementary multinomial logistic regression analysis conducted to examine factors associated with the intention to adopt a multidomain dementia prevention program, the “Considering adoption (timeline undecided)” group (*n* = 606) was treated as a separate outcome category, in addition to the “Active” (*n* = 110) and “Non-Adoption” (*n* = 197) groups. Compared to the Non-Adoption group, the Considering adoption group showed significant associations with indicators such as, prior experience with multidomain dementia prevention programs (RRR = 1.1; 95% CI: 1.0–1.3; *p* = 0.028), awareness of WHO-recommended prevention items (RRR = 1.2; 95% CI: 1.0–1.5; *p* = 0.048), and the number of currently implemented WHO-recommended items (RRR = 1.1; 95% CI: 1.0–1.2; *p* = 0.012). Other factors, including the implementation of measures to improve residents’ health literacy, did not show significant associations. Full results are presented in Additional file 1.

## Discussion

This nationwide cross-sectional survey of the Japanese municipalities aimed to clarify the current landscape of dementia prevention activities and identify factors that predict the adoption of comprehensive multidomain programs. Our analysis yielded two primary findings. First, we found that current municipal prevention efforts are predominantly concentrated on a combination of cognitive, social, and physical activity programs, with substantially less focus on the management of specific cardiovascular and lifestyle risk factors. Second, municipality’s intention to adopt a multidomain program was not significantly associated with its structural capacity, such as infrastructure or internal coordination. Instead, adoption intention was strongly predicted by the municipality’s programmatic readiness—defined by prior experience, awareness of evidence-based guidelines, and the number of prevention items already implemented—and its efforts to improve residents’ health literacy. These findings offer a quantitative perspective of the dementia prevention landscape at the municipal level in Japan and suggest that a municipality’s commitment to evidence-based practice and public health education are key drivers for adopting broader prevention strategies [6]. 

To situate these findings within major dementia-prevention frameworks, the 2024 Lancet Commission identifies 14 potentially modifiable risk factors across the life course and estimates that addressing them could prevent or delay a substantial proportion of dementia cases [7, 8]. In addition, the WHO Guidelines on Risk Reduction of Cognitive Decline and Dementia provide evidence-based recommendations targeting key behavioral and cardiometabolic risks, including physical activity, tobacco and alcohol use, healthy diet and weight management, and management of hypertension, diabetes, dyslipidemia, depression, and hearing loss [7]. 

Our survey operationalized dementia-prevention activities using 12 WHO-recommended items that map onto several established modifiable risk factors. However, we did not directly assess actions related to certain Lancet Commission factors (e.g., early-life education, air pollution, traumatic brain injury prevention, or vision loss), and the dyslipidemia item did not specifically distinguish elevated LDL cholesterol. From this perspective, multidomain interventions may be conceptualized as implementation packages that integrate multiple risk-reduction components, such as lifestyle modification, cognitive and social stimulation, and vascular risk monitoring, within a single program model. Nevertheless, effective population-level prevention is also likely to require complementary, cross-sectoral strategies addressing upstream determinants and late-life sensory health pathways. Besides these established frameworks, several candidate risk factors (e.g., sleep-related factors and other chronic exposures) continue to be investigated. However, the evidence base remains heterogeneous and has not yet been consistently incorporated into population-level policy frameworks.

Importantly, while trials such as FINGER have demonstrated statistically and clinically significant improvements in cognitive performance, these effects do not equate to complete prevention of dementia, and the magnitude of benefit varies across cognitive domains and participant subgroups. Subsequent multidomain trials conducted in diverse populations have yielded heterogeneous findings, underscoring that intervention effects are context-dependent and influenced by baseline risk profiles, adherence, and sociocultural environments. These considerations reinforce the need for population-level planning. Rather than viewing multidomain interventions solely as individualized clinical programs, they should be embedded within broader public health strategies aligned with local epidemiology and designed to shift risk distributions across entire communities.

Our first key finding was that municipalities in Japan predominantly implement a combination of cognitive, social, and physical activity programs, while placing less emphasis on the management of specific cardiovascular and lifestyle risk factors. This implementation pattern likely reflects practical and structural challenges. On a practical level, programs promoting social engagement and physical activity are often more straightforward for community centers to organize compared to interventions requiring specialized medical expertise and equipment [36, 37]. Furthermore, this may reflect a common organizational milieu within Japanese municipalities, where dementia prevention efforts, typically led by the long-term care division, are often separate from cardiovascular risk management programs, which fall under the health promotion division [38]. This siloing of services makes offering a truly integrated intervention difficult. Indeed, research on the implementation of comprehensive prevention programs in other countries has highlighted the lack of multiprofessional collaboration between different healthcare sectors as a significant barrier [16–19]. The observed pattern in Japan thus diverges from evidence-based models like those of the FINGER trial [5, 11], underscoring the necessity of combining lifestyle components with rigorous cardiovascular risk monitoring [39–41]. This suggests that to close the implementation gap, strategies must support individual program components and actively promote structural integration and collaboration between the health and long-term care departments within municipal governments.

In addition, municipal prioritization may reflect how dementia prevention has been framed in public discourse and local policy agendas. In Japan, dementia has received sustained attention as a national priority within long-term care policy and public messaging and has been widely portrayed as a major social and caregiving challenge in an aging society. This high visibility may incentivize municipalities to implement observable community-based activities (e.g., cognitive, social, and physical programs) that can be delivered through existing long-term care prevention platforms. By contrast, cardiovascular risk management is typically embedded within broader non-communicable disease strategies and clinical care pathways, rendering dementia-specific framing and program ownership less explicit at the municipal level. These differences in policy positioning may partially explain why cognitive and social programs appear more prominent than cardiovascular interventions in the present findings. These interpretations are exploratory and warrant further investigation, including through qualitative or mixed-methods studies.

Our second key finding reveals that a municipality’s intention to adopt a multidomain program was not predicted by structural factors like infrastructure or staffing levels, but rather by its programmatic readiness and efforts to improve resident health literacy. This suggests that the adoption of complex, evidence-based interventions is less about an organization’s static capacity and more about its dynamic state of preparedness and engagement. The strong association with prior experience and awareness of WHO guidelines points toward an evidence-based policy making orientation [7], where municipalities already familiar with such concepts are more likely to move towards implementation [42, 43]. 

In interpreting these findings, differences in municipal size and resource availability warrant careful consideration. Larger municipalities may benefit from greater administrative capacity, specialized personnel, and established intersectoral partnerships, which can facilitate the planning and adoption of complex multidomain dementia prevention programs. In contrast, smaller municipalities and rural towns may face workforce and organizational constraints, limiting their ability to adopt resource-intensive interventions, even when motivation and awareness are present. Although population size, staffing levels, and the number of community general support centers were included as control variables, contextual differences may still influence both adoption processes and survey response patterns. These factors should therefore be considered when interpreting the findings, particularly regarding their generalizability across municipalities with varying levels of resources.

Another important consideration is the relationship between local policy readiness and the epidemiological distribution of dementia risk factors across regions. Although population-level risk profiles—such as the prevalence of cardiovascular risk factors, hearing loss, or social isolation—are known to vary geographically, this study did not directly link municipal prevention policies with regional epidemiological data. Consequently, it was not possible to determine whether municipalities facing higher population-level dementia risk were more likely to adopt multidomain prevention strategies. Integrating policy data with regional epidemiological indicators represents an important direction for future research and would enable a more comprehensive evaluation of whether dementia prevention efforts are aligned with local risk profiles.

Importantly, epidemiological variability also has direct implications for policy prioritization and implementation strategy. Municipalities with a high burden of cardiometabolic risk may benefit from integrating dementia prevention into existing non-communicable disease programs, whereas areas characterized by social isolation or sensory impairment among older adults may require greater emphasis on social participation or hearing-related interventions. From this perspective, multidomain prevention should not be viewed as a fixed package applied uniformly across settings, but rather as a flexible framework adaptable to local epidemiological conditions. Programmatic readiness may interact with various factors, including available resources and managerial capacity, as discussed above in relation to municipal size. While geographical variations in risk are consistent with decentralized implementation, a multidomain approach requires either the concentration or collaboration of different sectors within an organization. Locally perceived risk burdens can influence how resources are allocated and how coordinated efforts are made in configuring prevention strategies.

Notably, the strongest predictor for adoption intention was the implementation of measures to improve residents’ health literacy. This can be interpreted in three ways. Firstly, by actively educating residents, municipalities may foster a community that better understands the importance of prevention, creating public demand and support for new programs [44]. Secondly, and perhaps more critically, these outreach activities enhance the capabilities of the public health workforce itself [45]. Efforts to create clear, accessible health information build the skills and confidence of municipal staff, which in turn makes them more prepared to tackle complex new interventions. This is supported by research in Japan showing that health literacy training for public health nurses was directly associated with increased confidence and subsequent application of new health promotion activities at the municipal level [46]. The development of health-literate materials through participatory methods further exemplifies this synergy, empowering both patients and providers [47]. Thirdly, the health literacy item may reflect the municipality’s motivation to implement new strategies. Research on health literacy in Japan only increased after 2000 [48]. In general, promoting new evidence and technologies in community planning is recommended [37]. Therefore, our findings suggest that fostering a culture of evidence-informed practice and investing in health literacy initiatives may be more effective for promoting the uptake of new programs than focusing on structural resources alone.

To further explore adoption readiness beyond the binary classification, we conducted a supplementary multinomial logistic regression analysis that included the “Considering adoption (timeline undecided)” group. This group comprised approximately two-thirds of respondents and showed significant associations with several indicators of programmatic readiness, including prior experience with multidomain interventions, awareness of WHO recommendations, and the number of implemented prevention items. These associations were generally weaker than those observed in the Active-Adoption group, suggesting a lower—but not absent—level of readiness. Given the large size and intermediate nature of this group, it likely includes municipalities with emerging interest and those with persistent indecision. Differentiating between this heterogeneous group and the clearly non-adoptive municipalities will be essential for designing tailored implementation strategies to encourage progression toward active adoption.

The study findings convey broad policy implications for Japan and any jurisdiction where regional or local governments hold the mandate for preventive health, suggesting the need to move beyond the uniform, one-size-fits-all approaches [49, 50]. Our results indicate that simply providing resources may be inadequate to drive the adoption of complex multidomain programs. Instead, a more effective national strategy would involve tailored, phased support that matches the programmatic readiness of each municipality. For municipalities not yet ready to adopt, initial support should focus on foundational capacity-building. This includes providing health literacy training for public health staff to enhance their skills and confidence, and fostering structural collaboration between health promotion and long-term care departments to mitigate organizational silos [46]. This approach aligns with the principles of complex intervention, which emphasize the importance of understanding and adapting to the local context and process rather than merely focusing on outcomes [51]. For municipalities that are “active” and demonstrate high readiness, support can then shift toward the technical assistance required to implement comprehensive, evidence-based programs. This type of multi-faceted, tailored implementation strategy is supported by systematic reviews, which reveal that combining multiple components is generally more effective than any single strategy [52]. Furthermore, facilitating collaboration between departments is a concrete way to realize the policy goal of integrating non-communicable disease management with dementia risk reduction efforts, ultimately creating a more efficient and effective pathway for the nationwide dissemination of updated dementia prevention.

This study has several limitations. First, although the response rate (54.4%) is relatively high for a national municipality survey in Japan, the possibility of response bias cannot be excluded. Municipalities more actively engaged in dementia prevention or with greater administrative capacity may have been more likely to participate, potentially leading to an overestimation of adoption intention and related practices. Indeed, responding municipalities demonstrated modestly higher fiscal capacity than non-respondents, suggesting a bias toward structurally stronger municipalities. Second, our primary analysis excluded municipalities that selected “Considering adoption (timeline undecided),” which accounted for approximately two-thirds of the sample. While this approach enabled clearer comparisons between municipalities with and without adoption intention, it limits generalizability. To address this concern, we conducted an exploratory multinomial regression analysis including this category see Additional file), thereby broadening the interpretability of the findings. Third, this survey focused specifically on initiatives implemented within the dementia prevention policy framework. However, some municipalities may deliver similar activities under broader long-term care prevention policies without explicitly labeling them as dementia-specific. Therefore, our analysis may not fully capture the entire scope of relevant municipal efforts. Furthermore, although this survey assessed policy orientation and implementation status at the municipal level, it did not measure participation rates or resident-level uptake. Accordingly, our findings reflect municipal policy positioning rather than the reach or effectiveness of implemented programs at the population level. Finally, we classified municipalities using administrative size categories rather than a standardized measure of rurality. Future studies should incorporate validated urban–rural indicators to better examine contextual differences in adoption and implementation.

## Conclusion

This nationwide survey of Japanese municipalities revealed that current dementia prevention efforts are concentrated on accessible lifestyle programs, while the adoption of more comprehensive, evidence-based interventions is predicted by programmatic readiness and health literacy outreach, not by structural capacity alone. At the same time, multidomain interventions should not be viewed as a single universal model. Although landmark trials such as FINGER provide important evidence, their effects vary across populations and do not represent dementia prevention completely. Effective implementation therefore requires adaptation to local epidemiological conditions, resource contexts, and community priorities. A tailored, population-informed approach will be essential for translating multidomain dementia prevention into sustainable public health practice.

## Supplementary Information

Supplementary Material 1.

Supplementary Material 2.

Supplementary Material 3.

## Data Availability

The data supporting the findings of this study are available from the corresponding author upon reasonable request.
