# Supplementary material for: Programmatic readiness and health literacy predict the adoption of multidomain dementia prevention: a nationwide survey of Japanese municipalities
Source: BMC Public Health. 2026 Jun 5;26:2371. doi: 10.1186/s12889-026-28068-8 (PMC13476930; doi:10.1186/s12889-026-28068-8)
Supplement: Supplementary file 1 [file 12889_2026_28068_MOESM1_ESM.docx]

Supplemental Table 1. Multinomial logistic regression results of factors associated with adoption intention

|  | Active vs. Non-Adoption (RRR, 95% CI) | *p*-value | Consideration vs. Non-Adoption (RRR, 95% CI) | *p*-value |
| --- | --- | --- | --- | --- |
| Prior experience with implementing a multidomain dementia prevention program | 1.43 (1.21-1.69) | <.001 | 1.13 (1.01-1.26) | 0.028 |
| Awareness of the WHO’s recommended dementia prevention items | 1.41 (1.02-1.95) | <.001 | 1.21 (1.00-1.46) | 0.048 |
| Number of WHO-recommended dementia prevention items implemented (per item) | 1.18 (1.07-1.30) | <.001 | 1.11 (1.02-1.20) | 0.012 |
| Implementation of measures to improve residents’ health literacy | 2.76 (1.18-6.45) | 0.019 | 1.43 (0.82-2.52) | 0.212 |
| Development of infrastructure, intersectoral collaboration, and cultural foundation | 0.62 (0.27-1.42) | 0.255 | 0.85 (0.49-1.47) | 0.552 |
| Availability of follow-up or post-program opportunities for participants | 1.25 (0.79-1.99) | 0.345 | 0.73 (0.54-0.99) | 0.048 |
| Level of coordination across departments within the municipal government | 0.96 (0.58-1.60) | 0.883 | 1.06 (0.76-1.49) | 0.716 |
| Degree of community and resident engagement in program planning or implementation | 1.09 (0.52-2.26) | 0.822 | 1.15 (0.70-1.88) | 0.582 |
| RRR: Relative risk ratio; CI: confidence interval. Estimates were derived using multinomial logistic regression models. All models were adjusted for municipality population size, aging rate, number of staff in charge of dementia-related policies, and number of community general support centers. For the variable "Number of WHO-recommended dementia prevention items implemented," the RRR represents the change associated with each additional item implemented. | | | | |
