# Supplementary material for: Programmatic readiness and health literacy predict the adoption of multidomain dementia prevention: a nationwide survey of Japanese municipalities
Source: BMC Public Health. 2026 Jun 5;26:2371. doi: 10.1186/s12889-026-28068-8 (PMC13476930; doi:10.1186/s12889-026-28068-8)
Supplement: Supplementary file 2 [file 12889_2026_28068_MOESM2_ESM.docx]

Additional file 1. Multinomial logistic regression results of factors associated with adoption intention

|  | Active vs. Non-Adoption (RRR, 95% CI) | *p*-value | Consideration vs. Non-Adoption (RRR, 95% CI) | *p*-value |
| --- | --- | --- | --- | --- |
| Prior experience with implementing a multidomain dementia prevention program | 1.4 (1.2-1.7) | <.001 | 1.1 (1.0-1.3) | 0.028 |
| Awareness of the WHO’s recommended dementia prevention items | 1.4 (1.0-2.0) | <.001 | 1.2 (1.0-1.5) | 0.048 |
| Number of WHO-recommended dementia prevention items implemented (per item) | 1.2 (1.1-1.3) | <.001 | 1.1 (1.0-1.2) | 0.012 |
| Implementation of measures to improve residents’ health literacy | 2.8 (1.2-6.5) | 0.019 | 1.4 (0.8-2.5) | 0.212 |
| Development of infrastructure, intersectoral collaboration, and cultural foundation | 0.6 (0.3-1.4) | 0.255 | 0.9 (0.5-1.5) | 0.552 |
| Availability of follow-up or post-program opportunities for participants | 1.3 (0.8-2.0) | 0.345 | 0.7 (0.5-1.0) | 0.048 |
| Level of coordination across departments within the municipal government | 1.0 (0.6-1.6) | 0.883 | 1.1 (0.8-1.5) | 0.716 |
| Degree of community and resident engagement in program planning or implementation | 1.1 (0.5-2.3) | 0.822 | 1.2 (0.7-1.9) | 0.582 |
| RRR: Relative risk ratio; CI: confidence interval. Estimates were derived using multinomial logistic regression models. All models were adjusted for municipality population size, aging rate, number of staff in charge of dementia-related policies, and number of community general support centers. For the variable "Number of WHO-recommended dementia prevention items implemented," the RRR represents the change associated with each additional item implemented. | | | | |
