# Supplementary material for: Programmatic readiness and health literacy predict the adoption of multidomain dementia prevention: a nationwide survey of Japanese municipalities
Source: BMC Public Health. 2026 Jun 5;26:2371. doi: 10.1186/s12889-026-28068-8 (PMC13476930; doi:10.1186/s12889-026-28068-8)
Supplement: Supplementary file 3 [file 12889_2026_28068_MOESM3_ESM.docx]

Figure 1.

Figure 2.

Figure 3.
